# Effect of cobalt ions on TNF-α and IL-6 secretion by fibroblasts surrounding hip periprosthetic membrane

**DOI:** 10.3389/fbioe.2025.1651049

**Published:** 2025-09-16

**Authors:** Ying Cai, Ang Li, Yebin Qian

**Affiliations:** Department of Orthopedic Surgery, Shanghai Sixth People’s Hospital Affiliated to Shanghai Jiao Tong University School of Medicine, Shanghai, China

**Keywords:** fibroblast, Co^2+^, glycolysis, aseptic loosening, proinflammatory cytokines

## Abstract

**Aims:**

The periprosthetic fibroblast-like cells (PPFs) play an important role in aseptic loosening after total hip arthroplasty (THA). However, little is known about fibroblast metabolism in aseptic loosening. Proinflammatory cytokines such as tumor necrosis factor-α (TNF-α) and il-6 interleukin-6 (IL-6) are involved in periprosthetic osteolysis. Cobalt (Co) ions are capable of inducing cytokines from macrophage. In this study, we investigated the effects of Co^2+^ on glycolysis and secretion of TNF-α and IL-6 in PPFs.

**Materials and methods:**

Fibroblasts were isolated from synovial tissues of osteoarthritis (OA) and rheumatoid arthritis (RA) patients, as well as from the periprosthetic pseudomembrane of patients undergoing revision surgery for aseptic loosening. Cells were cultured with or without Co^2+^. Following treatment, fibroblast viability was assessed using the MTT assay. To evaluate glycolysis, glucose uptake and lactate secretion were measured using specific assay kits. Furthermore, gene expression of key glycolysis enzymes (glucose transporter −1(GLUT1), hexokinase-2(HK2)) was analyzed by quantitative real-time PCR (qPCR), while protein expression of protein kinase B (AKT) and phosphorylated AKT (pAKT) was detected via Western blotting. Finally, TNF-α and IL-6 secretion into the culture supernatant was quantified using enzyme-linked immunosorbent assay (ELISA) kits.

**Results:**

Increased glucose uptake and lactic acid secretion occurred in PPFs. Exposure to Co^2+^ significantly increased glucose uptake, lactate secretion, GLUT1/HK2 mRNA expression, and TNF-α/IL-6 levels in PPFs. This Co^2+^-induced enhancement of glycolysis and cytokine secretion was dependent on glycolytic activity, as inhibition with 2-deoxy-D-glucose (2-DG) reduced all measured parameters. Furthermore, Co^2+^ stimulation increased pAKT protein expression in PPFs, indicating activation of the PI3K/AKT pathway. Consistent with this, treatment with the phosphatidylinositol three kinase/protein kinase B (PI3K/AKT) inhibitor LY294002 attenuated the Co^2+^-induced increases in glucose uptake, lactate secretion, GLUT1/HK2 mRNA, and TNF-α/IL-6 levels.

**Conclusion:**

Our findings suggest that Co^2+^ enhances TNF-α and IL-6 secretion in PPFs by upregulating glycolysis. This glycolytic regulation of cytokine production appears to be mediated by the PI3K/AKT signaling pathway, identifying it as a potential novel therapeutic target for preventing aseptic loosening.

## 1 Introduction

Joint arthroplasty is by far the most effective treatment for many end-stage joint diseases such as rheumatoid arthritis and osteoarthritis. Prosthesis failure can occur for many reasons, such as aseptic loosening, prosthesis damage, periprosthetic fracture, infection and dislocation. One study found that of 803 patients undergoing revision after primary THA, 535 (66.6%) underwent revision due to aseptic loosening, indicating that aseptic loosening is the primary reason of revision THA (Feng et al., 2022). The pathogenesis of aseptic loosening is very complex, and the pathogenesis includes the biological mechanism and mechanical mechanism. Multiple cell types contributed to the occurrence of aseptic loosening, such as macrophages, osteoblasts, osteoclasts and fibroblasts. Although macrophages and osteoclasts play a dominant role in aseptic loosening, studies have shown that other cells, such as fibroblasts, also have an impact on this process (Yao et al., 1995; Wei et al., 2009). PPFs are the predominant cell types in the periprosthetic pseudomembrane. PPFs have been shown to play a significant role in aseptic loosening (Pap et al., 2003). Recent evidence highlights that fibroblast activation in this context is not merely a passive response but involves a profound metabolic reprogramming towards glycolysis, akin to the Warburg effect observed in activated immune cells. This glycolytic shift is believed to provide the rapid energy and biosynthetic precursors necessary for the heightened secretory and proliferative functions of activated fibroblasts, including the production of cytokines and matrix-remodeling enzymes (Bustamante et al., 2017; Weichhart et al., 2015).

There were high levels of proinflammatory factors in the periprosthetic membrane (Chiba et al., 1994; Shanbhag et al., 1995). TNF-α and IL-6 are particularly important (Roodman et al., 1992; Tamura et al., 1993; Inoue et al., 2000; Azuma et al., 2000). Titanium (Ti) particle stimulation increased the secretion of proinflammatory cytokines TNF-α, IL-6, IL-8 and IL-1β in human fibroblasts (Yang et al., 2021; Sharma et al., 2020). The microenvironment caused by these pro-inflammatory cytokines intensifies bone resorption, resulting in aseptic loosening.

Research related to metal-on-metal (MoM) joint prostheses has been revived because of its adverse reaction. MoM joint prostheses result in early revision rates (Huang D. et al., 2013), unexplained pain (Campbell et al., 2008) and high blood metal ion levels (Cobb and Schmalzreid, 2006; Hart et al., 2009). Studies have shown that biological adverse reactions associated with high metal ion concentrations include bone loss, local soft tissue toxicity, and inflammation (Shimmin et al., 2005; Hailer et al., 2011). Interestingly, cobalt concentrations as high as 30 μM were detected in the synovial fluid from the failed MoM hip prosthesis (Kwon et al., 2011). Co^2+^ ions are a powerful cellular stress mediator in synovial fibroblasts. In synovial fibroblasts, mitochondrial stress, pro-inflammatory responses, and activation of hypoxia are stimulated by Co/Cr, resulting in the release of chemokines, growth factors, cytokines, and other molecules that may trigger inflammation of the periprosthetic tissues through leukocyte recruitment and endothelial activation (Eltit et al., 2021).

Carbohydrate metabolism, as a basic biochemical process, mainly ensures that living cells can obtain energy continuously. Glucose, the most important carbohydrate, is transported to cells first by the GLUT1, then decomposed into pyruvate by a series of metabolic enzymes, including hexokinase (HK), phosphoglycerate kinase (PGK1), aldolase and pyruvate kinase, and finally will either be converted to lactate by lactate dehydrogenase (LDH) or will enter tricarboxylic acid (TCA) cycle and oxidative phosphorylation to produce ATP. Glycolysis plays a role in the pathogenesis of aseptic loosening. Blocking the glycolysis pathway has been shown to inhibit osteoclast generation, suggesting that glycolysis plays a key role in osteoclast differentiation (Regan et al., 2014). Upregulation of glycolysis was detected after stimulation of macrophages with metal ions (Indo et al., 2013). However, the regulation and mechanism of glycolysis in PPFs remain unclear. The PI3K/AKT signaling pathway is associated with glycolysis. The activation of PI3K/AKT signaling pathway can promote the occurrence and development of inflammatory response, which leads to the exacerbation of the inflammatory response in the periprosthetic tissue and further accelerates the occurrence of aseptic loosening. Recent work has specifically linked PI3K/AKT signaling to metabolic reprogramming in stromal cells within inflammatory environments, positioning it as a master regulator of cellular metabolism and inflammation (Weichhart et al., 2015; Fruman et al., 2017).

These considerations led us to investigate changes in glycolysis in PPFs as well as glycolysis and secretion of TNF-α and IL-6 from PPFs under Co^2+^ stimulation. We further investigated the effect of blocking glycolysis on the secretion of TNF-α and IL-6 from PPFs using a glycolysis inhibitor, 2-DG. Finally, we investigated whether the PI3K/AKT signaling pathway is involved in glycolytic metabolism in regulating the secretion of TNF-α and IL-6 from Co^2+^ stimulated PPFs.

## 2 Materials and methods

### 2.1 Patients

Specimens of the periprosthetic pseudomembrane were collected from eight patients undergoing revision surgery for aseptic loosening of THA. The indication for primary THA in all patients with aseptic loosening was OA. All patients showed aseptic loosening with radioactive osteolysis. No patients showed signs of clinical infection. During the THA, we collected synovial tissues from 10 patients with OA according to American College of Rheumatology criteria. We excluded patients with other chronic inflammatory diseases, immunological abnormalities, trauma, or surgery. Synovium was derived from 12 RA patients who received THA. All RA patients met the criteria for seropositive RA as revised by the American College of Rheumatology 1987. Patients with other chronic inflammatory diseases, immunological abnormalities, trauma, or surgery were not included. All procedures were approved from the institutional ethical committee.

### 2.2 Cell culture

Tissue was collected from patients as described above, washed several times with phosphate-buffered saline (PBS), cut into small pieces, and digested with α-Minimal Essential Medium (α-MEM) (Gibco, Life Technologies, CA, United States) containing 1 mg/mL collagenase (Sigma-Aldrich, St. Louis, MO) for 1 h at 37 °C. The completely digested tissue was then filtered through a 70 μm cell strainer. Then, the digested tissue was centrifuged (1,500 rcf, 10 min). The pellet was resuspended in α-MEM supplemented with 60 IU/mL penicillin, 60 μg/mL streptomycinand 10% fetal bovine serum (FBS) (Sorfa, Beijing, China), and cultured in a T75 culture flaskat 37 °C and 5% CO_2_. The medium was changed twice a week. Cells were passaged at 80%–90% confluence by using 0.05% trypsin(Gibco, Life Technologies, CA, United States of America). Afterwards, cells were used in passage between three and eight for the following assay.

### 2.3 Western blot analysis

The periprosthetic fibroblast-like cells were disrupted in lysis buffer. Proteins were separated by SDS-PAGE and transferred to a nitrocellulose membrane. Blots were probed with p-AKT (Cell Signaling Technology, Beverly, MA) and total AKT (Cell Signaling Technology Beverly, MA) and actin (Beyotime, Shanghai, China) at 1:1,000 dilution. Horseradish peroxidase-conjugated anti-IgG (Beyotime, Shanghai, China) was used as secondary antibody at 1:2,000 dilution. Membranes were developed using a chemiluminescence system.

### 2.4 ELISA

IL-6 and TNF-α from supernatants were evaluated by enzyme-linked immunosorbent assay(Multi Sciences, Hangzhou, China) following the manufacturer’s protocol.

### 2.5 MTT assay

For the MTT assay, 3 × 10^3^ PPFs/well were plated into 96-well plates in 10% FBS/DMEM. After 24 h, the medium was replaced with low-serum medium (0.1% FBS/DMEM) for 24 h for synchronization. On day 0, medium was replaced with 1% FBS and cells were treated with 2DG (50 mM) or medium without glucose. Co^2+^ or PBS alone was added to the appropriate wells. Cell viability was estimated on day 4 after incubation with MTT for 4 h and was read at 550 nm with a spectrophotometer.

### 2.6 Real-time quantitative PCR (qPCR)

The periprosthetic fibroblast-like cells were collected and total RNA was extracted with Total RNA Extraction Reagent Kit (EZBioscience, Roseville, the United States). RNA was quantified and assessed for purity using a NanoDrop spectrophotometer. Total RNA 200 ng from each sample was used for cDNA synthesis using the Color Reverse Transcription Kit (EZBioscience, Roseville, the United States). qPCR was performed with SYBR Green qPCR Master Mix Kit (EZBioscience, Roseville, the United States). The relative amounts of transcripts were compared to those of HRPT and normalized to untreated samples by the ^∆∆^Ct method. Primers are available upon request.

### 2.7 Lactate and glucose measurement

Media samples stimulation were collected after 24 h of Co^2+^ and stored at −20 °C until the time of the assay. Glucose assay kit(Beyotime, Shanghai, China) and L-lactate assay kit(Boxbio Science & Technology Co., Beijing, china) were measured in the conditioned media of cell incubations using colorimetric kits according to manufacturer’s instructions.

### 2.8 Statistical analysis

Statistical analyses were conducted using Prism software. Data are presented as mean ± SEM. Variable normality was assessed using the Shapiro-Wilk test and D’Agostino Pearson tests. For comparison between two groups, student’s two-tailed t-tests (parametric) or Mann-Whitney tests (nonparametric) were used, based on distribution normality. For comparisons among three or more groups, one-way analysis of variance (ANOVA) and two-way ANOVA were performed. Depending on the homogeneity of variances, either Dunnett’s *post hoc* test or the Bonferroni correction was applied. Statistical significance was defined as a two-sided P value <0.05.

## 3 Results

### 3.1 Glucose metabolism in the periprosthetic fibroblast-like cells

**FIGURE 1:**
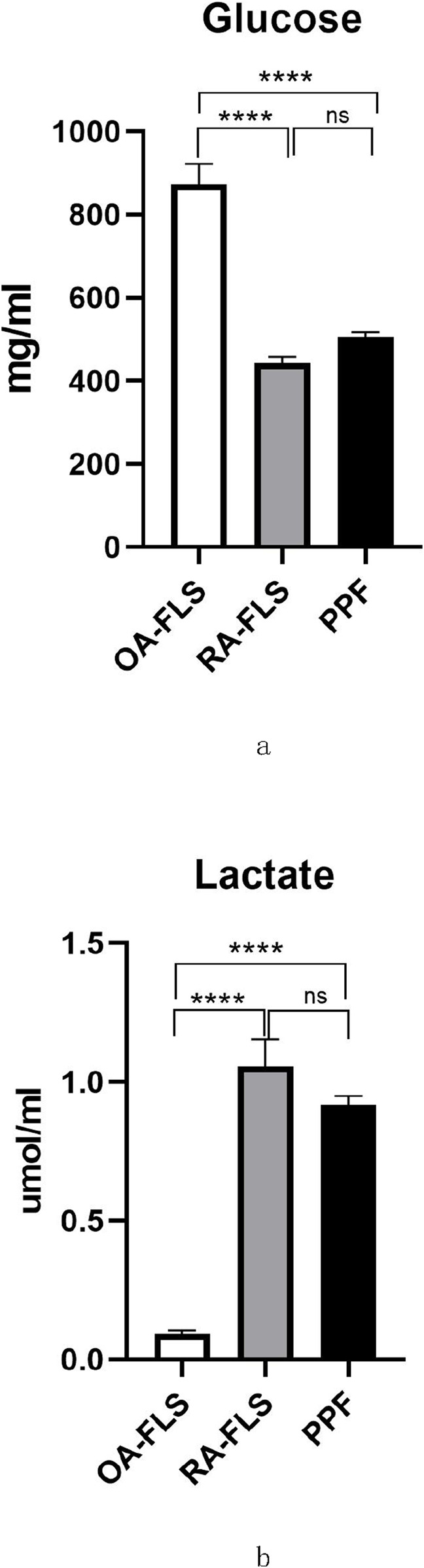
Glucose and lactate levels. After passaging the cells for 4 times, we used glucose assay kit and lactate assay kit to measure glucose **(a)** and lactate **(b)** levels. Results in a–b are pooled from three different cell lines. Values are the mean ± SEM. ** = P < 0.05; *** = P < 0.01; **** = P < 0.001.

We first measured the glucose and lactate in the supernatant of the cultured primary periprosthetic fibroblast-like cells to investigate whether PPFs showed an increase in the metabolism of glucose to lactate. Using glucose assay kit and lactate assay kit, we measured glucose and lactate levels in supernatant of RA fibroblast-like cells, OA fibroblast-like cells and periprosthetic fibroblast-like cells (Figures 1a,b). The glucose level of periprosthetic fibroblast-like cells was lower than OA fibroblast-like cells and was similar RA fibroblast-like cells. However, PPFs showed a higher lactate value than OA fibroblast-like cells and was comparable to RA fibroblast-like cells. Studies have shown that RA fibroblast-like cells are upregulated in glycolysis. To some extent, PPFs demonstrated an increase in the metabolism of glucose to lactate.

### 3.2 Effects of Co^2+^ on glycolysis and function of the periprosthetic fibroblast-like cells

**FIGURE 2:**
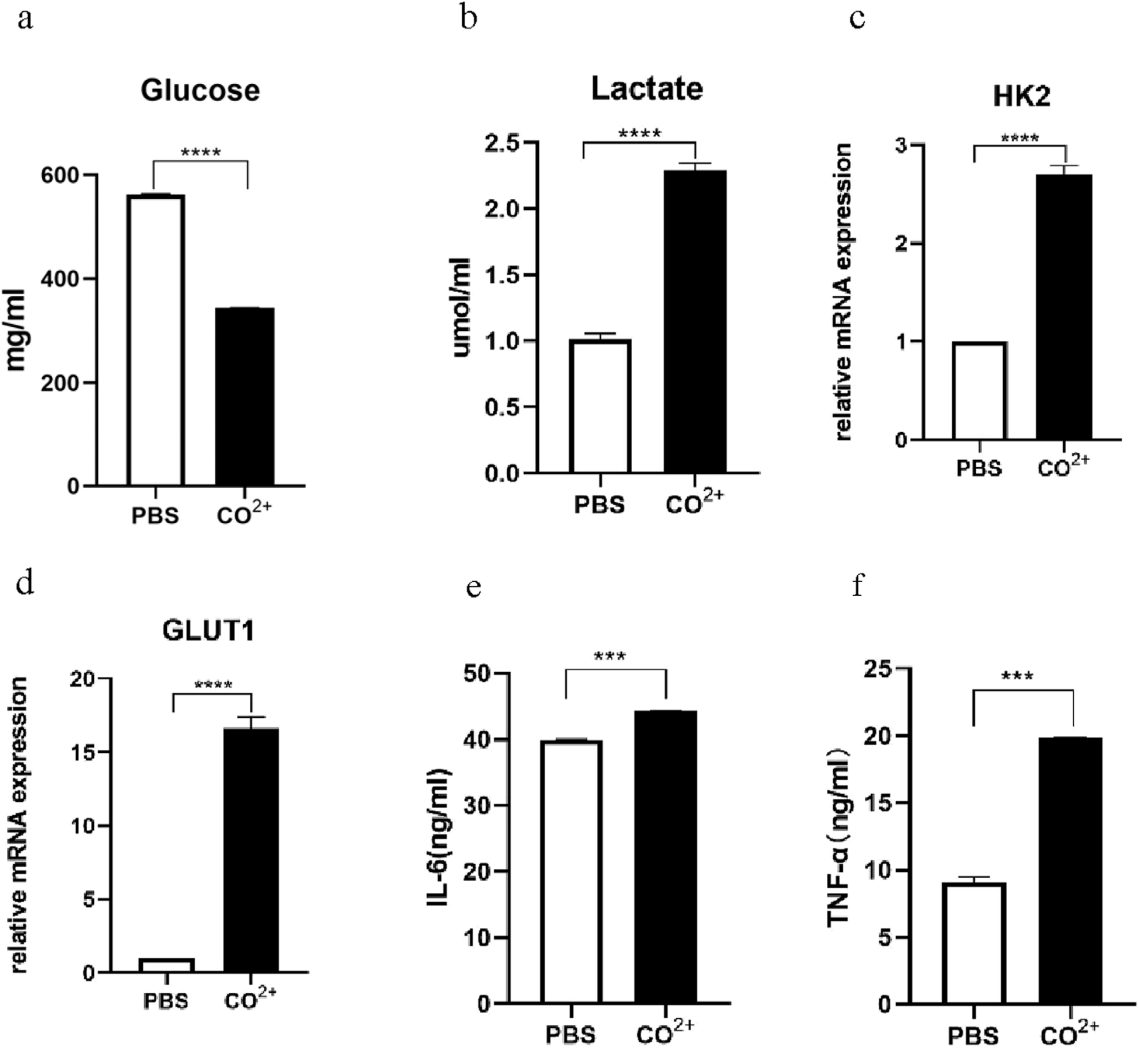
Glycolysis and secretory function of PPFs after Co^2+^ stimulation. **(a,b)**, the periprosthetic fibroblast-like cells were stimulated with Co^2+^ or phosphate-buffered saline (PBS) as vehicle control, for 24 h, followed by measurement of the Glucose **(a)** and Lactate **(b)**. **(c,d)**, the periprosthetic fibroblast-like cells were stimulated with Co^2+^ or phosphate-buffered saline (PBS) as vehicle control, for 24 h, the expression of messenger RNA (mRNA) for HK2 **(c)** and GLUT1 **(d)** were determined by quantitative polymerase chain reaction (qPCR). **(e,f)**, Supernatants from PPFs cultures were prepared after 24 h of Co^2+^ stimulation and were analyzed for secretion of IL-6 **(e)** and TNF-α **(f)**. Results in a–g are pooled from three different cell lines. Values are the mean ± SEM. *** = P < 0.01; **** = P < 0.001.

To determine whether Co^2+^ regulates glycolysis and secretion functions of PPFs, PPFs were stimulated with Co^2+^ for 24 h. Co^2+^ stimulation of PPFs stimulates glycolysis (Figures 2a,b). To further investigate the effect of Co^2+^ on glycolysis, we stimulated cells with Co^2+^ and identified the expression of messenger RNA (mRNA)for GLUT1 and HK2 by quantitative polymerase chain reaction (qPCR). As shown in Figures 2c,d, the expression of enzymes closely related to glycolysis significantly increased after Co^2+^ stimulation. Then, tumor necrosis factor-α(TNF-α) and interleukin-6 (IL-6) in supernatant of Co^2+^ stimulation for 24h were detected by ELISA, and Co^2+^ stimulation significantly increased TNF-α and IL-6 level as compared to the level in the control cells (Figures 2e,f).

### 3.3 Effects of inhibition of glycolysis on the periprosthetic fibroblast-like cells function

**FIGURE 3:**
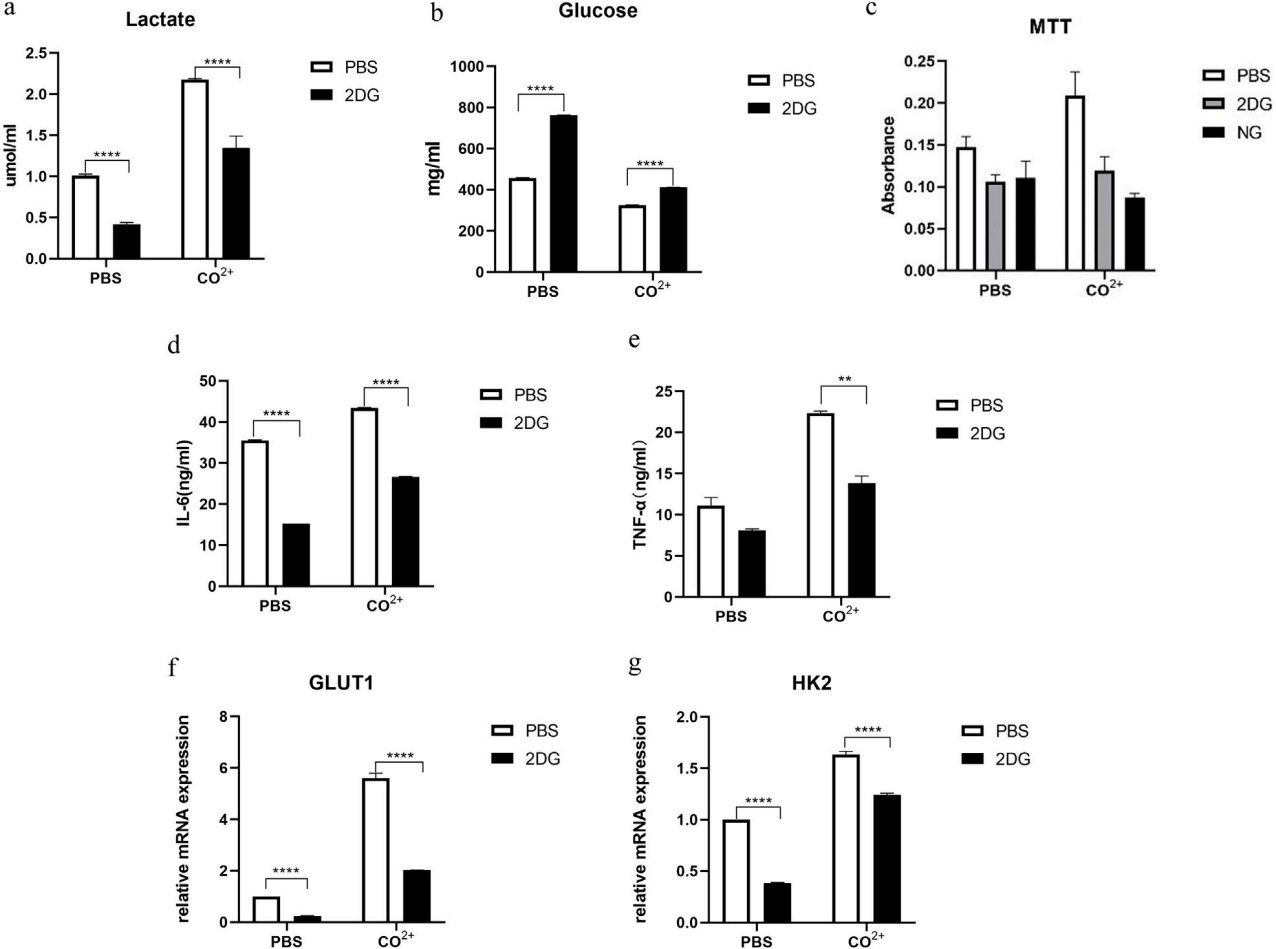
effect of inhibition of glycolysis on the periprosthetic fibroblast-like cells function. **(a,b)** **(a)** Lactate and **(b)** glucose in the supernatant 24 h after Co^2+^ or PBS stimulation in the presence or absence of glycolysis inhibitors (2-DG: 50 mM in PBS). **(c)** The periprosthetic fibroblast-like cells were cultured in the presence of Co^2+^ or PBS, with or without 2-DG (50 mM in PBS) or no glucose (NG) medium. Cellular proliferation was determined by MTT assay on day 4. **(d,e)** In the presence of Co^2+^ or PBS as carrier control, the PPFs were cultured with or without 2DG (50 mM) pretreatment. Supernatant from cell cultures was prepared after 24 h of Co^2+^ stimulation and were analyzed for secretion of IL-6 **(d)** and TNF-α **(e)**. **(f,g)** the periprosthetic fibroblast-like cells were stimulated with Co^2+^ or PBS), with or without 2-DG (50 mM in PBS), the mRNA for GLUT1 **(f)** and HK2 **(g)** were determined by qPCR. Results in a–g are pooled from three different cell lines. Values are the mean ± SEM. ** = P < 0.05; **** = P < 0.001.

Since glycolysis was stimulated by Co^2+^ treatment of cells, we determined whether inhibition of glycolysis during Co^2+^ exposure could alter the response of PPFs to the factor. Glucose consumption and lactate secretion after LPS stimulation were first measured in the presence or absence of inhibitor. As expected, incubation in PPFs with 2DG prevented increase in lactate levels and decrease in glucose levels in response to Co^2+^ (Figures 3a,b).

Next, we determined whether inhibition of glycolysis might interfere with PPFs growth *in vitro*. We used 2-deoxyglucose (2-DG), a glucose analogue that is phosphorylated to phospho-2-DG by HK but cannot be further metabolized by phospho-glucose isomerase. As an alternative approach to limiting glycolysis, we also investigated the effect of growing PPFs in glucose deficient medium (no glucose [NG] control medium). The PPFs was pretreated with 2-DG or NG control medium, and then cultured in the presence of Co^2+^ for 4 days, followed by MTT assay. MTT analysis showed that the cell proliferation rate was significantly reduced under the two culture conditions (Figure 3c).

Treatment with 2-DG also reduced the secretion of IL-6 and TNF-α in PPFs (Figures 3d,e). In addition, mRNA expression of GLUT1 and HK2 which are associated with glycolysis dramatically decreased in PPFs pretreated with 2DG (Figures 3f,g).

### 3.4 Role of PI3K/AKT pathway in increased glycolysis and functional changes of Co^2+^ stimulated the periprosthetic fibroblast-like cells

We further investigated signaling pathways that may play a role in increased glycolysis and functional changes of periprosthetic fibroblasts stimulated by Co^2+^ PI3K/AKT signaling pathway is involved in biological processes such as cell apoptosis, cell cycle, angiogenesis and glucose metabolism. We used PI3K-Akt inhibitor, LY294002, to target PPFs.

**FIGURE 4:**
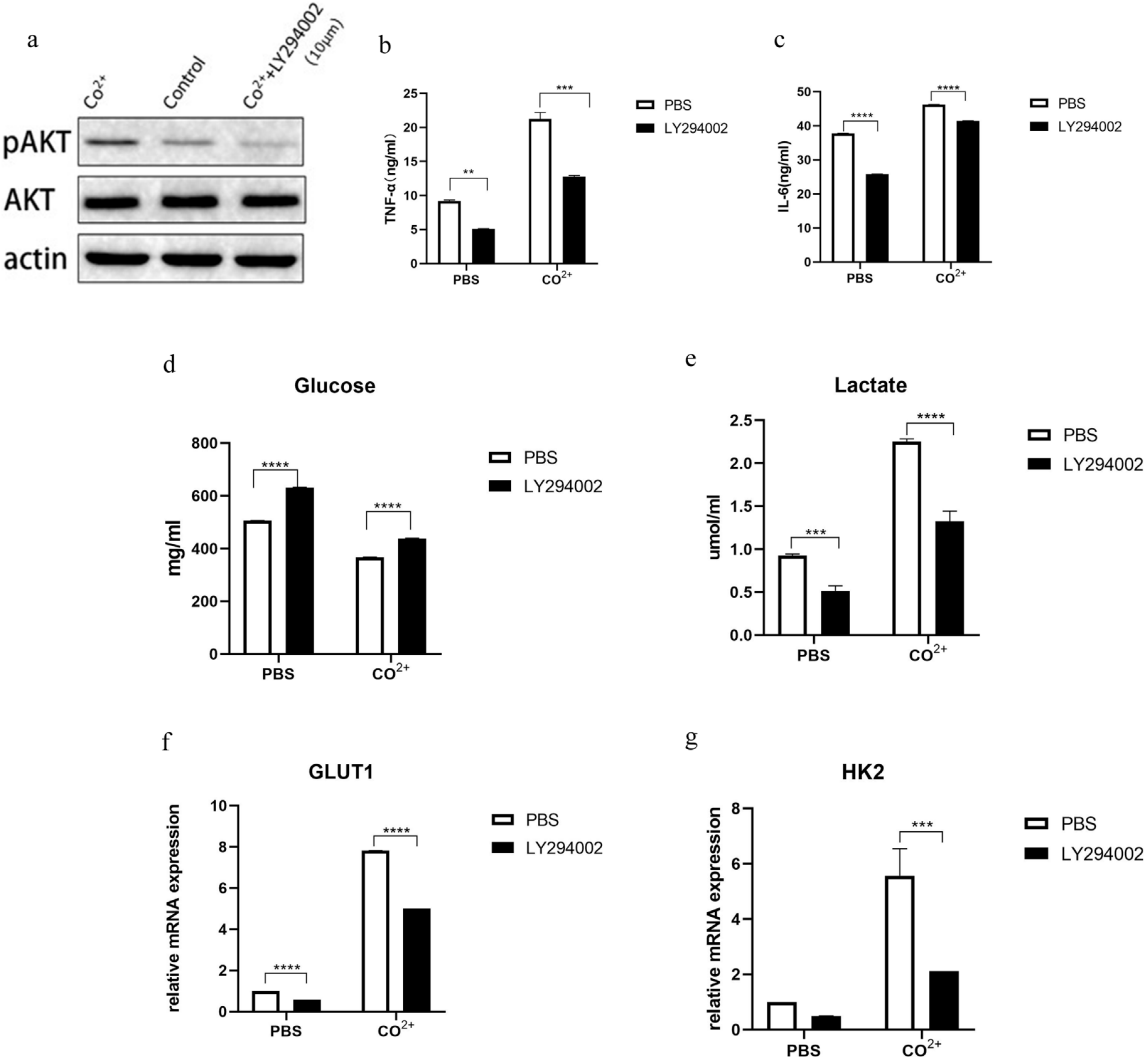
Role of PI3K/AKT pathway in increased glycolysis and functional changes of Co^2+^ stimulated periprosthetic fibroblast-like cells. **(a)** The periprosthetic fibroblast-like cells (n = 3 cell lines) was first incubated with an PI3K inhibitor (LY294002) at different concentrations for 1 hour and then stimulated with Co^2+^ or PBS for 15 min. P-AKT, AKT and actin expression was determined my WB. **(b,c)** In the presence or absence of inhibitors, Supernatant from cell cultures was prepared and the secretion of IL-6 **(b)** and TNF-α **(c)** was analyzed. **(d,e)** In the presence or absence of inhibitors, glucose **(d)** and lactate **(e)** in supernatant after Co^2+^ or PBS stimulation for 24 h. **(f,g)** The periprosthetic fibroblast-like cells were cultured with or without LY294002. The mRNA expression of GLUT1 **(f)** and HK2 **(g)** was determined by qPCR. Results in b–g are pooled from four different cell lines. Values are the mean ± SEM. *** = P < 0.01; **** = P < 0.001.

First, PPFs were incubated with different concentrations of LY294002, and then p-AKT and AKT were measured by WB (Figure 4a). A follow-up study was conducted with a concentration of 10 μM. As shown in Figure 4a, Co^2+^ stimulation significantly increased AKT phosphorylation compared to the control group.

Next, we determined whether this pathway is involved in the secretion of PPFs. The secretion of IL-6 and TNF-α decreased after LY294002 treatment (Figures 4b,c). At the same time, we determined whether this pathway was associated with changes in glucose metabolism in periprosthetic fibroblasts after Co^2+^ stimulation. Glucose consumption and lactate secretion were first measured in the presence or absence of PI3K inhibitors, LY2924002. After the use of inhibitors, glucose levels (Figure 4d) in PPFs increased, while lactate levels (Figure 4e) decreased. Indicating that glycolysis is inhibited. Interestingly, GLUT1 and HK2, enzymes associated with glycolysis, were decreased (Figures 4f,g).

## 4 Discussion

Macrophages, fibroblasts, osteoblasts, osteoclasts and lymphocytes were involved in the development of aseptic loosening. The roles of macrophages, osteoblasts and osteoclasts have been extensively studied. Wear particles stimulate macrophages to produce chemokines such as chitinase 1 (CHIT1), C-C motif chemokine 18(CCL18), IL-8 and macrophage inflammatory protein 1α (MIP1α), forming a chemokine environment conducive to recruitment and maturation of osteoclast progenitors, and stimulating osteoclast maturation through a sharp decline in osteoprotegerin (OPG) levels (Koulouvaris et al., 2008). Macrophages phagocytic bone cement particles produce specific mediators that may be tumor necrosis factor to stimulate the secretion of prostaglandin E2 (PGE2) by osteoblasts, which then magnifies the inflammatory response, leading to bone resorption and aseptic loosening (Horowitz et al., 1994). A small amount of bacterial components and metal wear particles together induce enhanced inflammatory responses in human monocytes and osteoblasts, and this effect can significantly promote the production of bone resorption osteoclasts, leading to implant loosening (Chen et al., 2021). Fibroblast is the main cell type in the synovial tissue formed around the loosening of prosthesis. Ti particles induce the expression of pro-inflammatory cytokines in periprosthetic membrane-derived fibroblasts (Yang et al., 2021). Ti particles or substance P stimulated the expression of Receptor Activator of Nuclear Factor-κ B Ligand (RANKL) and cyclooxygenase −2(COX-2) in fibroblasts around the prostheses (Qian et al., 2013). Increased expressions of collagen and stromatolysin were found after stimulation of the fibroblasts from patients with failed total hip replacement using titanium particles, suggesting that wear granule-stimulated fibroblasts may play an important role in periprosthetic osteolysis through the release of bone resorption-metalloproteinases and mediators (Yao et al., 1995). However, studies of fibroblasts have not been fully elucidated. In patients with rheumatoid joints, glucose levels in inflammatory synovium are low, while lactate levels are high (de Oliveira et al., 2019). Increased glucose uptake in rheumatoid arthritis fibroblasts after tumor necrosis factor stimulation (Ahn et al., 2015). Another study found that glucose metabolism in fibroblasts in rheumatoid arthritis shifted to a glycolytic pathway (Garcia-Carbonell et al., 2016). This immunometabolic phenotype, characterized by a glycolytic switch supporting pro-inflammatory activation, is increasingly recognized as a key driver of pathogenic inflammation and tissue remodeling in chronic diseases (Bustamante et al., 2017; Weichhart et al., 2015). In this study, we found that glycolysis was upregulated in PPFs, indicating that glycolysis may play a role in aseptic loosening.

Wear particles are one of the key biological factors affecting the service life of artificial joints, and their existence often leads to the failure of surgery. Different prosthesis materials will produce different wear particles. With the evolution and development of prosthesis materials, wear particles such as polyethylene particles, bone cement particles, titanium alloy particles, cobalt-chrome-molybdenum particles, Co^2+^ and chromium (Cr) ion are produced. A study showed that the polyethylene wear particles caused the proliferation of synovium and stimulated macrophages and multinucleated giant cells (Howie et al., 1993). Wear and corrosion on the titanium alloy surface lead to the release of debris, which can lead to osteolysis and implant loosening. When stimulated by titanium dioxide nanoparticles (TiO2 NPs), exosomes were secreted in osteoblasts and reduced osteogenic differentiation of human mesenchymal stem cells (de Souza et al., 2023). Cobalt-chromium-molybdenum alloy particles, titanium particles, zirconia and Zr mixtures can stimulate the secretion of TNF-α, IL-6 and IL-8 from fibroblasts, osteoblasts and monocytes/macrophages (Dalal et al., 2012). The wear particles stimulate the secretion of various inflammatory mediators in the synovial tissue surrounding the loosening of the prosthesis. Cytokines such as IL-6, IL-1, TNF-α and prostaglandin E2 activate osteoclasts, cause osteolysis and aseptic loosening of artificial joints. TNF-α and IL-6 play an important role in aseptic loosening (Roodman et al., 1992; Tamura et al., 1993; Inoue et al., 2000; Azuma et al., 2000; Fuller et al., 2002). TNF-α directly induced the formation of mature osteoclasts - Tartrate resistant acid phosphatase-positive multinucleated cells (TRAP + MNCs) and played an significant role in local osteolysis in chronic inflammatory diseases (Azuma et al., 2000). Studies have shown that IL-6 promotes osteoclast generation and bone resorption (Kudo et al., 2003). Titanium particles stimulated the secretion of pro-inflammatory cytokines TNF-α, IL-6, IL-8 and IL-1β in human fibroblasts (Yang et al., 2021; Sharma et al., 2020). Studies have shown that cobalt ions stimulate mouse macrophages to secrete pro-inflammatory cytokines TNF-α and IL-6 (Yang et al., 2022). Cobalt ions induce mouse microglia to release inflammatory mediators and upregulate the production of pro-inflammatory cytokines TNF-α and IL-6 (Mou et al., 2012). Co^2+^ stimulates the secretion of TNF-α and IL-6 from osteoarthritis fibroblasts (Eltit et al., 2021). Histological studies of failed MoM implants have consistently demonstrated necrotic and inflammatory changes in periprosthetic tissues associated with elevated metal ion levels (Mahendra et al., 2009). In our study, we found that the secretion of TNF-α and IL-6 increased in the PPFs treated with Co^2+^. This suggests that Co^2+^ stimulates the secretion of TNF-α and IL-6 from PPFs.

Glycolysis is one of the main ways that cells use glucose. Glucose is transported into the cell via GLUT1 located on the cell membrane. In the cytoplasm, glucose is metabolized to pyruvate by a series of glycolytic enzymes such as HK2. The expression of glycolytic related genes HK2 and GLUT1 was upregulated in osteoarthritis fibroblasts stimulated by Co^2+^, while no glycolytic related genes were upregulated in Cr^3+^-stimulated osteoarthritis fibroblasts (Eltit et al., 2021). Co^2+^ (but not Cr^3+^) induces macrophages to transition from oxidative phosphorylation to HIF-1α-dependent glycolysis, this metabolic change may play an early and critical role in the Co^2+^ induced inflammatory response in the periprosthetic environment (Salloum et al., 2021). In our study, we found that glucose uptake and lactate secretion and mRNA expression of GLUT1 and HK2 were enhanced in PPFs under Co^2+^ stimulation. These indicate that the glycolysis of PPFs was upregulated under Co^2+^ stimulation, and Co^2+^ is a potent stimulator of glycolysis in PPFs.

Studies have found that macrophages can enhance Hypoxia-inducible Factor1-α(HIF1-α) gene expression and protein stability even in aerobic environment, promote glucose intake and lactic acid synthesis by up-regulating target genes such as glucose transporter and lactate dehydrogenase, thus leading to increased glycolysis level, and promote the transcription of inflammation related genes such as TNF, IL-6 and IL-1Ra (Mascanfroni et al., 2015; Boutens et al., 2018; Tai et al., 2009). The rise of IL-1β in M1 macrophages can be effectively blocked by a glycolytic inhibitor,2-DG (Tannahill et al., 2013). Treg expresses the transcription factor Foxp3, secretes IL-10, inhibits inflammation and maintains immune tolerance. Th17 cells express transcription factor RORγt and secrete IL-17, which plays a key role in inducing inflammation and autoimmune diseases (Cluxton et al., 2019). Glycolysis inhibitor 2-DG not only promoted the differentiation of Treg cells, but also inhibited the differentiation of Th17 cells and induced Foxp3 expression, while fatty acid oxidation inhibitor inhibited the differentiation of Treg cells (Shi et al., 2011; Shi and Chi, 2019; Maciolek et al., 2014). These studies indicate that glycolysis is related to the occurrence of inflammation and secretion of inflammatory factors. We found that Co^2+^ stimulated PPFs produced less TNF-α and IL-6 after inhibition of glycolysis. These results suggest that Co^2+^ may upregulate the secretion of TNF-α and IL-6 in PPFs through glycolysis.

Glycolysis is associated with a variety of signaling pathways. The PI3K/AKT signaling pathway is associated with glycolysis. The protein expression of glycolytic enzyme decreased after LY294002 treatment, while the number of mitochondria and mitochondrial membrane potential increased. The key parameters of extracellular acidification rate decreased significantly after inhibiting PI3K/AKT signaling pathway, and the key parameters of oxygen consumption rate increased significantly. The PI3K/AKT pathway has been shown to promote proliferation and inhibit apoptosis of keloid fibroblasts under hypoxia by regulating glycolysis (Wang et al., 2023). PI3K/AKT signaling pathway is not only activated in inflammatory synovial tissue, but also leads to the occurrence and development of inflammatory response (Huang J. B. et al., 2013). The PI3K/AKT/mTOR axis is a well-established master regulator that integrates metabolic and inflammatory signals in various immune and stromal cells (Fruman et al., 2017; Xie et al., 2019). In our study, pAKT protein expression was increased in Co^2+^ stimulated PPFs, while glycolysis and TNF-α and IL-6 secretion were decreased in Co^2+^ stimulated PPFs treated with PI3K/AKT signaling inhibitor LY294002. Theses suggest that Co^2+^ regulates glycolysis and the secretion of TNF-α and IL-6 in PPFs through PI3K/AKT signaling pathway.

### 4.1 Potential confounders and study limitations

While our *in vitro* model provides valuable insights into the specific effects of Co^2+^ on PPF metabolism and cytokine secretion, we acknowledge several potential confounders that may limit the direct translation of these findings to the *in vivo* environment. Our system utilizes isolated fibroblasts exposed to a single metal ion (Co^2+^) at a specific concentration. *In vivo*, PPFs exist within a complex multicellular milieu, interacting directly with macrophages, lymphocytes, osteoblasts, and osteoclasts. These interactions, mediated by cell-cell contact and paracrine signaling, can profoundly modulate fibroblast activation (Rao et al., 2012). Furthermore, the periprosthetic environment contains a mixture of wear debris (polyethylene, titanium, cobalt-chromium alloy particles) and metal ions (Co^2+^, Cr^3+^, Ti^4+^), which may act synergistically or antagonistically. The absence of mechanical stress, a critical factor in aseptic loosening that influences both cell signaling and cytokine production, is another significant limitation of our static culture model (Brodbeck et al., 2003). Finally, our study represents an acute exposure model, while aseptic loosening is a chronic process occurring over years. It is imperative to account for this patient and environmental variability when evaluating implant failure. Future studies employing co-culture systems, patient-derived tissue explants, and *in vivo* models will be essential to validate these findings in a more physiologically relevant context.

### 4.2 Therapeutic implications and translational potential

Our findings identify the PI3K/AKT-glycolysis axis in PPFs as a potential novel therapeutic target for mitigating inflammation-driven osteolysis. Translating this into a clinical strategy would require highly localized drug delivery to avoid systemic toxicity, as PI3K/AKT and glycolysis are fundamental pathways in nearly all cells. Systemic inhibition would be highly toxic, causing immunosuppression, impaired wound healing, and other adverse effects (Fruman and Rommel, 2014). Potential approaches could include developing implant coatings or biodegradable hydrogels that elute specific PI3K/AKT or glycolytic inhibitors (e.g., isoform-specific PI3K inhibitors or 2-DG analogues) directly into the periprosthetic space (Gulati et al., 2012). This would aim to disrupt the pathogenic metabolic programming of PPFs and other innate immune cells without affecting systemic metabolism. However, significant safety considerations must be addressed. Even with local delivery, off-target effects on surrounding bone cells are a concern; for instance, inhibiting glycolysis could potentially impair osteoblast function and bone formation (Lee et al., 2017). Therefore, any therapeutic strategy would require exquisitely targeted delivery and a careful balance between inhibiting pathological inflammation and preserving normal tissue homeostasis and repair processes. Extensive *in vivo* efficacy and safety studies are mandatory before any clinical application can be contemplated.

## 5 Conclusion

In our study, we found that glycolysis was upregulated and the secretion of TNF-α and IL-6 increased in PPFs stimulated by Co^2+^. Blocking glycolysis could inhibit the secretion of TNF-α and IL-6 in fibroblasts. Therefore, Co^2+^ may increase the secretion of TNF-α and IL-6 by PPFs through upregulation of glycolysis. Inhibition of PI3K/AKT signaling pathway led to downregulation of glycolysis and decreased secretion of TNF-α and IL-6 in PPFs under Co^2+^ stimulation, suggesting that Co^2+^ regulates glycolysis and the secretion of TNF-α and IL-6 in PPFs through PI3K/AKT signaling pathway. We believe that further research on the role and mechanism of glycolysis in fibroblasts will provide a new direction for the prevention and treatment of aseptic loosening.

## Data Availability

The raw data supporting the conclusions of this article will be made available by the authors, without undue reservation.
