# Supplementary material for: Effect of cobalt ions on TNF-α and IL-6 secretion by fibroblasts surrounding hip periprosthetic membrane
Source: Front Bioeng Biotechnol. 2025 Sep 16;13:1651049. doi: 10.3389/fbioe.2025.1651049 (PMC12479478; doi:10.3389/fbioe.2025.1651049)
Supplement: Supplementary file 1 [file Supplementaryfile1.docx]

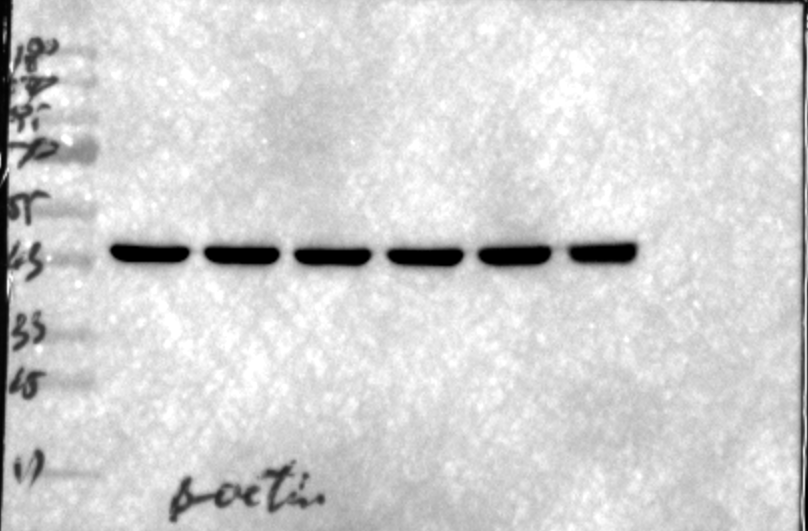


S1. Unprocessed raw image for actin detection by Western blot


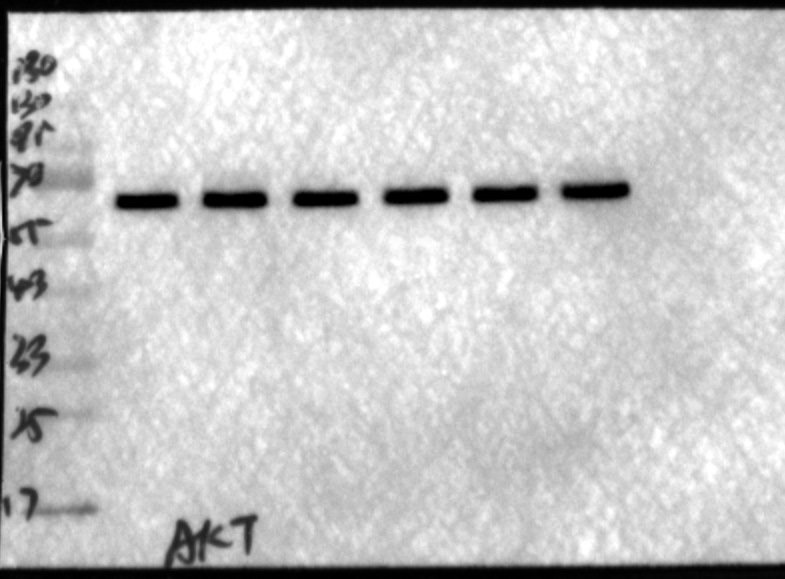


S2. Unprocessed raw image for AKT detection by Western blot


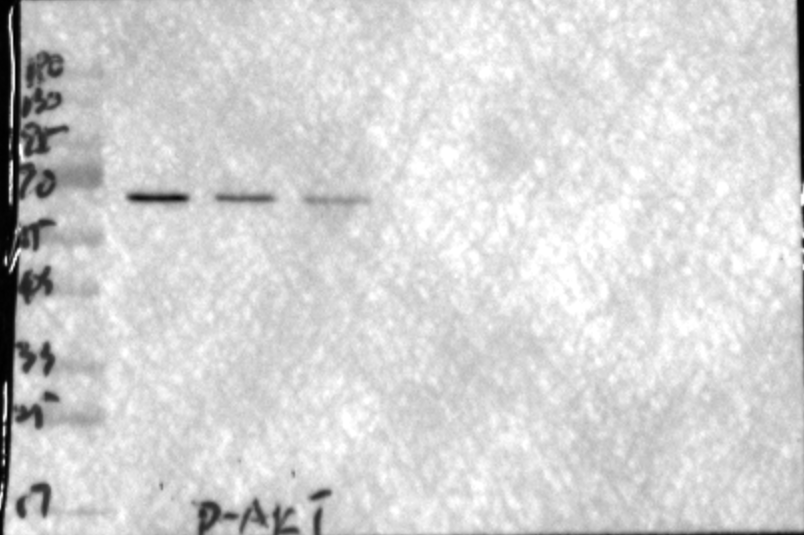


S3. Unprocessed raw image for pAKT detection by Western blot
